# A comparison of urinary tract pathology and morbidity in adult populations from endemic and non-endemic zones for urinary schistosomiasis on Unguja Island, Zanzibar

**DOI:** 10.1186/1471-2334-9-189

**Published:** 2009-11-29

**Authors:** Beatrice Lyons, Russel Stothard, David Rollinson, Simba Khamis, Khamis A Simai, Paul R Hunter

**Affiliations:** 1 School of Medicine, Health Policy and Practice, University of East Anglia, Norwich, UK; 2 Wolfson Wellcome Biomedical Laboratories, Zoology Dept, The Natural History Museum, London, UK; 3 Helminth Control Laboratory Unguja, Ministry of Health and Social Welfare, Zanzibar, Tanzania; 4 Mnazi Moja Hospital, of Health and Social Welfare, Zanzibar, Tanzania

## Abstract

**Background:**

Renal tract involvement is implicated in both early and late schistosomiasis leading to increased disease burden. Despite there being good estimates of disease burden due to renal tract disease secondary to schistosomiasis at the global level, it is often difficult to translate these estimates into local communities. The aim of this study was to assess the burden of urinary tract pathology and morbidity due to schistosomiasis in Zanzibar and identify reliable clinical predictors of schistosomiasis associated renal disease.

**Methods:**

A cross-sectional comparison of Ungujan men and women living within either high or low endemic areas for urinary schistosomiasis was conducted. Using urine analysis with reagent strips, parasitological egg counts, portable ultrasonography and a qualitative case-history questionnaire. Data analysis used single and multiple predictor variable logistic regression.

**Results:**

One hundred and sixty people were examined in the high endemic area (63% women and 37% men), and 101 people in the low endemic area (61% women and 39% men). In the high endemic area, egg-patent schistosomiasis and urinary tract pathology were much more common (p = 1 × 10^-3^, 8 × 10^-6^, respectively) in comparison with the low endemic area. Self-reported frothy urine, self-reported haematuria, dysuria and urgency to urinate were associated with urinary tract pathology (p = 1.8 × 10^-2^, p = 1.1 × 10^-4^, p = 1.3 × 10^-6^, p = 1.1 × 10^-7^, respectively) as assessed by ultrasonography. In a multi-variable logistic regression model, self-reporting of schistosomiasis in the past year, self-reporting of urgency to urinate and having an egg-positive urine sample were all independently associated with detectable urinary tract abnormality, consistent with schistosomiasis-specific disease. Having two or more of these features was moderately sensitive (70%) as a predictor for urinary tract abnormality with high specificity (92%).

**Conclusion:**

Having two out of urgency to urinate, self reporting of previous infections and detection of eggs in the urine were good proxy predictors of urinary tract abnormality as detected by ultrasound.

## Background

After malaria, schistosomiasis is the second most prevalent and important parasitic disease in the world in terms of socio-economic impact [1]. Urinary schistosomiasis, caused by infection with *Schistosoma haematobium*, is a major health problem on the islands of Zanzibar (Unguja and Pemba) located adjacent to the mainland coast of East Africa [2]. Typically, schistosomiasis is a chronic infection causing significant morbidity. Even though the infection may have self-cleared parasitologically as evidenced by the cessation of egg excretion, disease sequelae may persist. Renal tract involvement is implicated in both early and late disease, leading to increased disease burden which is likely to impact activities of daily living and the countries' economy and welfare. Although there have been good attempts at estimating the totality of disease burden due to schistosomiasis at a global level [3], it is often difficult to translate these estimates into local communities, not least because of significant geographical heterogeneity of the disease set against other confounding factors.

Unguja island can be divided into 2 disease landscapes for urinary schistosomiasis - northern: the endemic zone of transmission where the intermediate host snail *Bulinus globosus *is found; southern: the non-endemic zone where *B. globosus *is absent and the refractory snail *Bulinus nasutus *is found [4]. This malacological division is the causal factor limiting the local distribution of urinary schistosomiasis in the human populace, ranging from less than 5% in the south (in Muyuni, Kizimkazi, Kiongoni Makunduchi and Paje) to over 60% in the north [5]. This strong epidemiological divide provides a convenient opportunity for the first comparative study of urinary tract pathology and disease morbidity since the work of Donald Forsyth on the island in the 1960s [6].

More recently, use of an assay to detect excreted albumin in urine has suggested that the onset of kidney disease and spontaneous kidney failure is associated with *S. haematobium *infection [7]. However, the extent of urinary tract damage caused by schistosomiasis in Zanzibar since the work of Forsyth in the 1960s [8] is still not clear. More broadly studies have used portable ultrasound to assess urinary tract pathology in Zimbabwe [9] and elsewhere [10]. Nevertheless, the relationship between urinary tract pathology and morbidity and schistosomiasis burden remains to be determined precisely. Most people do not experience advanced forms of disease, but instead suffer from less obvious symptoms such as anaemia, effects of stunted growth, lowered work capacity, abdominal pain, and exercise intolerance [11].

This study was undertaken to determine whether urinary tract pathology and morbidity still varied significantly between high and low schistosomiasis endemic areas and so to provide an estimate of burden of urinary tract pathology and morbidity due to schistosomiasis. In addition, the study was designed to improve our understanding of those clinical features that may be predictive of schistosomiasis associated renal disease.

## Methods

### Study area

Local drug dispensaries located in the main villages in the north (Chaani, Mwera, Upenja and Kinyasini) and in the south (Paje and Muyuni) were used as convenient stations to recruit members of the local populace and act as bases to perform portable ultrasonography. Each village was visited two days prior to the day of survey by staff of the Helminth Control Laboratory Unguja (HCLU) who would liaise with the local village leader (shehia) for community sensitization and assistance in open enrolment of future patient volunteers (> 16 years of age) for the day of survey. The survey took place during June and July 2007. Exclusion criteria were a history of recent travel outside the immediate area and any obvious current ill-health which would make attendance at the mobile clinic problematic. On the day of survey, each patient volunteer was provided with further information about schistosomiasis and requested to confirm their study assent in writing (or if illiterate by thumbprint after further discussion with HCLU staff) prior to answering the case-history questionnaire, undergoing the ultrasound scan and submitting their urine sample.

### Case-history questionnaire

The qualitative health related behaviour questionnaire used to determine disease burden was based on the SF-12 short form questionnaire (SF-12^® ^Health Survey^© ^1994). The questionnaire was split into categories: 'personal information', 'water contact', 'disease symptoms', 'ability to perform activities of daily living'. The patient information leaflet and questionnaires were discussed with and adapted by members of the HCLU field team in order to make them culturally acceptable. The questionnaire was then translated into Kiswahili and later back translated into English to cross check for accuracy of initial translation. Two female lab technicians assisted subjects with the questionnaires - the questionnaire in English and Kiswahili is available by request from corresponding author.

### Examination by ultrasound

To reduce observational bias, patients' parasitological status was blinded from the ultrasonographer, though the ultrasonographer was still aware whether they were working in a high or low risk area. Upper and lower urinary tracts were examined and observable pathology was classified according to the WHO Niamey criteria [1].

### Urine sample

Each urine sample was assessed for with reagent strips (Hemastix^®^, Bayer, UK). The number of schistosome eggs was ascertained by filtering a 10 ml aliquot through a 12 μm polycarbonate filter (Millipore, UK) which was viewed at ×100 under a light compound microscope according to WHO guidelines [12,13]. Although multiple urine samples may be required to confirm precisely the parasitological status of an individual, it was hoped that this level of parasitological sampling would be sufficient for a general comparison between endemic and non-endemic areas.

### Analysis

The study was powered to detect an absolute difference in incidence of disease between the two populations of 20% with a power of 80% and alpha of 5%. It was estimated that 107 volunteers were required from each group. Data analysis was undertaken with the statistical software package SPSS 10.0 Windows (SSPS Inc., Chicago, USA).

### Ethical consideration

Ethical approval was gained by the Zanzibar Health Research Council. Patient confidentiality was assured by assigning codes to the each ultrasound scan, urine sample and questionnaire responses. Praziquantel was only offered to participants who required treatment for egg-patent schistosomiasis. If there was identification of severe, untreatable kidney pathology (cancer), or any other abnormal abdominal ultrasound findings (i.e. fibroids) the participant was referred to the national hospital in Stone Town.

## Results

### Demographic data

A total of 160 people were surveyed in the north and 101 people were surveyed in the South, though no urine sample was received from four patients (three from the North and one from the South). The mean age of study participants was 32 years old (std deviation 14.7) and females made up 63%. There was no significant difference in the gender balance, age or Body Mass Index between volunteers from the North and the South. However, people in the north were more likely to have been to school than those in the south (p = 1.9 × 10^-6^) and more likely to be farmers (p = 6.4 × 10^-4^). The latter is not surprising given that the North is a predominantly agricultural area, whereas in the south the soils are much less fertile and generally poorer and fishing is a more likely occupation.

### Prevalence of markers of schistosomiasis

Indicators of schistosomiasis (haematuria, egg count and ultrasound evidence of urinary tract pathology) are shown in (Table 1) clearly demarcating the expected high and low endemicity. Most of the urinary tract pathology visualized on ultrasound was classifiable within the WHO criteria for schistosomiasis induced urinary tract pathology. However, other urinary tract pathologies which were identified included 3 cases of pyelonephritis, 2 cases of enlarged prostates, 1 case of duplicate kidneys, 1 case of bilateral polycystic kidney, 3 cases of uterine fibroid (the uterine fibroids were of interest as they can cause urinary symptoms). However, these cases were not counted as 'urinary tract pathologies' as the pathology could not clearly be related to schistosomiasis-specific sequelae.

**Table 1:** Epidemiological variation of schistosomiasis infection based on egg count, haematuria and urinary tract pathology (as seen on ultrasound) between north and south.

	South		North		P value
	N	% positive	N	% positive	
Eggs in urine	100	0	157	10.2^b^	0.001

Urinary tract pathology	101	1.0	156	17.3	0.00004

Haematuria detected on dipstick^a^	99	0	156	8.3	0.002

^a^Excluding menstruating women

^b^In one case eggs were non viable

### Clinical symptoms associated with urinary tract pathology

Objective and subjective clinical symptoms and signs were analyzed for area of residence and association with urinary tract pathology using (Table 2). Pain on micturition, urgency to urinate, self-perceived frothy urine and self-perceived haematuria were significantly associated with those living in the high endemic area and also with having urinary tract pathology detected on ultrasound. Loin pain and reporting being tired by mid-day was significantly associated with living in the high endemic area but not with the presence of urinary tract pathology.

**Table 2:** Incidence of clinical symptoms in endemic and non-endemic regions and their association with urinary tract pathology as determined by ultrasound examination.

Variable		Association with living in the South or North					Association with presence of urinary tract pathology (as detected on ultrasound)				
		
		S	N	Risk ratio	95% CI	*P *value	No	yes	Risk ratio	95% CI	*P *value
Breathlessness	No	81	120	1.26	0.79-2.04	0.4	177	21	1.10	0.54-2.03	0.7
	Yes	20	40				52	7			

Oedema	No	93	153	0.55	0.21-1.43	0.2	217	25	2.04	0.63-6.08	0.3
	Yes	8	7				12	3			

Pain on micturating	No	88	112	2.33	1.36-4.10	0.002	186	10	3.42	2.25-4.91	9 × 10^-8^
	Yes	13	48				43	18			

Frequency	No	90	141	1.09	0.55-2.18	0.8	205	22	2.04	0.90-4.26	0.1
	Yes	11	19				24	6			

Urgency	No	91	110	3.16	1.72-5.94	0.00008	188	9	3.79	2.52-5.41	0.00007
	Yes	10	50				41	19			

Loin pain	No	68	84	1.45	1.06-2.03	0.02	133	17	0.94	0.55-1.42	0.8
	Yes	33	76				96	11			

Tired by midday	No	81	97	2.01	1.30-3.18	0.001	162	15	1.55	0.93-2.35	0.1
	Yes	19	60				65	12			

Self perceived frothy urine	No	94	133	2.43	1.14-5.33	0.02	203	20	2.52	1.23-4.74	0.02
	Yes	7	27				26	8			

Self perceived haematuria	No	95	122	4.00	1.82-9,01	0.001	198	15	3.43	1.98-5.94	0.0002
	Yes	6	38				31	13			

Questions relating to the respondent's ability to perform activities of daily living included: how often in the past month each lacked the energy to perform activities for daily living, how often their health limited gentle activities and whether their health limited what they would like to do. None of these variables were associated with detected urinary tract pathology or living in the high endemic area. Neither was there any association with self-reported difficulty in conceiving or painful intercourse.

### Predictors of urinary tract pathology

In addition to the clinical symptoms discussed above, 71% of cases with pathology reported suffering from schistosomiasis in the past year compared to only 23% of people without pathology (p = 8 × 10^-8^). Also 39% of cases had detectable eggs in the urine compared to just 2% of those without pathology (p = 2 × 10^-14^). The corresponding figures for detectable haematuria were 26% and 3% (p = 2 × 10^-7^). Consequently the self-reporting of schistosomiasis in past year, eggs in urine and haematuria are all predictors of renal pathology.

Table 3 shows the specificity of the significant symptoms from Table 2 as predictors of urinary tract abnormality on ultrasound along with self reported schistosomiasis in the past year, eggs in the urine and detected haematuria. In this analysis self-reported schistosomiasis in the past year was the most sensitive 71% with specificity of 77%. In a logistic regression analysis of all these variables only urgency, self-reported schistosomiasis in the past year, and eggs in the urine were significantly associated with urinary tract abnormality. These three variables were combined to determine the sensitivity and specificity multiple symptoms (Table 3). Having all three of these features was highly specific 100%, but not very sensitive 22%, whereas having one or more was highly sensitive 93% but lower specificity 64%. Having two or more of these characteristics gives a reasonably sensitivity of 70%, whilst still a specificity of >90%. This analysis was re-run with the clinical symptoms of pain on passing urine and or urgency, but this did not improve sensitivity and reduced specificity.

**Table 3:** Sensitivity and specificity of symptoms and signs in predicting urinary tract abnormality on ultrasound.

	Sensitivity 95%CI (%)		Specificity 95%CI (%)	
Pain on micturating	64	44 -- 81	81	76 -- 86

Urgency	68	48 -- 84	82	77 -- 87

Self perceived frothy urine	29	13 -- 49	89	84 -- 92

Self perceived haematuria	46	28 -- 66	86	81 -- 91

Self reported schistosomiasis in past year	71	51 -- 87	77	71 -- 82

Detected haematuria	26	11 -- 46	97	94 -- 99

Detected eggs in urine	39	22 -- 59	98	95 -- 99

				

One or more of self-reported schistosomiasis in past year, urgency and eggs in urine	93	76-99	64	58 -- 71

Two or more of self-reported schistosomiasis in past year, urgency and eggs in urine	70	50 -- 86	92	88 -- 96

All three of self-reported schistosomiasis in past year, urgency and eggs in urine	22	9 -- 42	100	98 -- 100

## Discussion

This study compared the burden of schistosomiasis of people living in the north of the island (high endemicity) with those living in the south of the island (low endemicity) to assess the individual burden of disease. Whilst comparisons of this nature have been done in the past [6,10] it should be noted that even within the high endemic area there can be substantial variation in exposure to schistosomiasis and evolution of the disease at the individual level [2].

It is also worthy of note that the 'Kick-out-Kichocho' programme (which was launched in 2003) has been set up to provide annual administration of antihelmintics (albendazole and praziquantel) to all primary school children living within the high endemic zone [5]. Our study began 1 month after the 4^th ^cycle of treatment in schools and associated communities, and 65% of the participants in the north and 4% of those from the south reported to have taken an antihelmintic in the past year. Against this background of widespread use of antihelmintics, one would expect that the prevalence of infection would have fallen over the past few years amongst participants in the 'north cohort', even though the people in this study would have been too old to receive praziquantel as part of the Kick-out-Kichocho programme. Previous ultrasound studies suggest that regular antihelminth drug treatment can cause regression of pathology [14,15] and this may have also impacted upon our own findings. However, even with the treatment programme in place, this study has demonstrated substantially higher prevalence of infection, urinary tract pathology and symptoms amongst people living in the endemic region. The continued high prevalence of schistosomiasis in the North despite a regular campaign of antihelminthic is a cause for concern.

When set against historical data, the prevalence of infection and detected urinary tract abnormalities were lower than that reported in "bad areas" during the 1960s [6,8] suggesting that progress has been made in the control of this disease and its urological complications. However, van der Werf and de Vlas [16] showed that studies based in schools gave higher prevalence of detected urinary tract abnormality on ultrasound than community based studies. They suggested that this was probably an age related phenomenon, though the possibility remains that community studies may also fail to recruit the individuals with the most severe urinary tract abnormalities. However, a major explanation will be the different gender balance. In our study females made up the majority of participants and males were much more likely to report schistosomiasis in the past year than females (51% *vs *15%, p = 2 × 10^-9 ^Fishers exact test)

The finding that people living in endemic areas were more likely to be tired by midday supported a previous study showing that schistosomiasis can cause fatigue and reduce exercise tolerance [11], though in the study reported here there was no association with ultrasound detected urinary tract abnormalities, suggesting either that the increased fatigue reported in the North was not related to demonstrable urinary tract abnormality or was even due to other confounding factors. There are, of course a wide range of physical and psychological conditions that can predispose to fatigue [17] and so feeling tired is unlikely to be a very specific symptom for schistosomiasis.

Egg counts were performed on all the urine samples, though for most analysis presence-absence of eggs was used for analysis due to the small number of positive samples identified, thus intensity of infection was not specifically taken into consideration. However, a previous study suggested that there was no obvious trend between the intensity of infection (egg count) and bladder pathology [18]. It is thought that the limiting factor to urinary tract pathology is dependent on genetically determined immune regulation of the inflammatory response and not to the infection load [10,19]. Although volunteers with 'any signs of obvious ill health' were excluded, potential confounders were the participants who were found to have non-schistosomiasis related pathology on ultrasound that may have been causing them urinary symptoms. However, all the cases (pyelonephritis, enlarged prostate, polycystic kidney, duplicate kidney, uterine fibroids) where found in participants living in the non-endemic region.

Self-perceived frothy urine, self-perceived haematuria, dysuria and urgency to urinate were all associated with urinary tract pathology, especially dysuria and urgency. Although self perceived frothy urine, haematuria, dysuria and urgency are useful diagnostic tools, the effect of these clinical signs on daily performance is unclear. Frothy urine and haematuria may not be considered a burden to the individual, and thus these signs may not be a reason for seeking medical attention, nor mentioned to the health care provider. Therefore national educational programmes should ensure that people in endemic areas are aware of clinical signs to watch out for and when to report these to their doctor/dispensary staff. In a review of 21 papers, van der Werf *et al. *[20] reported good correlation with the general prevalence of infection in a community that did not seem to suffer from recall bias with varying recall times. However, in the study presented here self-reported haematuria was not a good predictor of urinary tract abnormality, indicating that markers of population prevalence are not always reliable diagnostic markers of infection in individual cases.

Previous studies have demonstrated that self reporting of illness is an effective way of identifying current schistosomiasis infection [20,21]. Our findings further suggest that self reported illness was a strong predictor of urinary tract pathology. Self reporting can be used as a simple clinical tool for diagnostic purposes. Furthermore the participants (both men and women) appeared to be very willing to volunteer information relating to urinary symptoms and previous schistosomiasis infection and there appeared to be no stigmatisation, or taboo, associated with the condition.

## Conclusion

Self perceived symptoms, especially urgency to urinate, self reporting of previous infections and detection of eggs in the urine are good predictors of urinary tract abnormality as detected by ultrasound. If two or more of these features are present in a patient then this has reasonably good diagnostic power for urinary tract abnormality and people should be investigated further. This finding is of clinical importance for local doctors as it can be used as a tool to identify individuals with potential urinary tract pathology for triage of further investigations.

## Competing interests

The authors declare that they have no competing interests.

## Authors' contributions

BL, JRS and DR conceived of the study. BL, JRS, DR and PRH participated in the design. BL, ISK and KAS undertook data collection. Statistical analysis was performed by PRH. BL and PRH wrote the first drafts but all authors contributed to writing the manuscript. All authors read and approved the final manuscript.

## Pre-publication history

The pre-publication history for this paper can be accessed here:

http://www.biomedcentral.com/1471-2334/9/189/prepub
